# Turning gray selenium and sublimed sulfur into a nanocomposite to accelerate tissue regeneration by isothermal recrystallization

**DOI:** 10.1186/s12951-023-01796-4

**Published:** 2023-02-21

**Authors:** Jieqiong Cao, Yibo Zhang, Yiqi Yang, Junye Xie, Zijian Su, Fu Li, Jingsheng Li, Bihui Zhang, Zhenyu Wang, Peiguang Zhang, Zhixin Li, Liu He, Hongwei Liu, Wenjie Zheng, Shuixing Zhang, An Hong, Xiaojia Chen

**Affiliations:** 1 Department of Cell Biology & Institute of Biomedicine, College of Life Science and Technology, Guangdong Province Key Laboratory of Bioengineering Medicine, Jinan University, Guangdong Provincial Biotechnology Drug & Engineering Technology Research Center, National Engineering Research Center of Genetic Medicine, Guangzhou, China; 2 The First Affiliated Hospital of Jinan University, Guangzhou, China; 3 Department of Chemistry, Jinan University, Guangzhou, China

**Keywords:** Nanocomposite, Tissue regeneration, Low toxicity, Nanoengineering

## Abstract

**Background:**

Globally, millions of patients suffer from regenerative deficiencies, such as refractory wound healing, which is characterized by excessive inflammation and abnormal angiogenesis. Growth factors and stem cells are currently employed to accelerate tissue repair and regeneration; however, they are complex and costly. Thus, the exploration of new regeneration accelerators is of considerable medical interest. This study developed a plain nanoparticle that accelerates tissue regeneration with the involvement of angiogenesis and inflammatory regulation.

**Methods:**

Grey selenium and sublimed sulphur were thermalized in PEG-200 and isothermally recrystallised to composite nanoparticles (Nano-Se@S). The tissue regeneration accelerating activities of Nano-Se@S were evaluated in mice, zebrafish, chick embryos, and human cells. Transcriptomic analysis was performed to investigate the potential mechanisms involved during tissue regeneration.

**Results:**

Through the cooperation of sulphur, which is inert to tissue regeneration, Nano-Se@S demonstrated improved tissue regeneration acceleration activity compared to Nano-Se. Transcriptome analysis revealed that Nano-Se@S improved biosynthesis and ROS scavenging but suppressed inflammation. The ROS scavenging and angiogenesis-promoting activities of Nano-Se@S were further confirmed in transgenic zebrafish and chick embryos. Interestingly, we found that Nano-Se@S recruits leukocytes to the wound surface at the early stage of regeneration, which contributes to sterilization during regeneration.

**Conclusion:**

Our study highlights Nano-Se@S as a tissue regeneration accelerator, and Nano-Se@S may provide new inspiration for therapeutics for regenerative-deficient diseases.

**Graphical Abstract:**

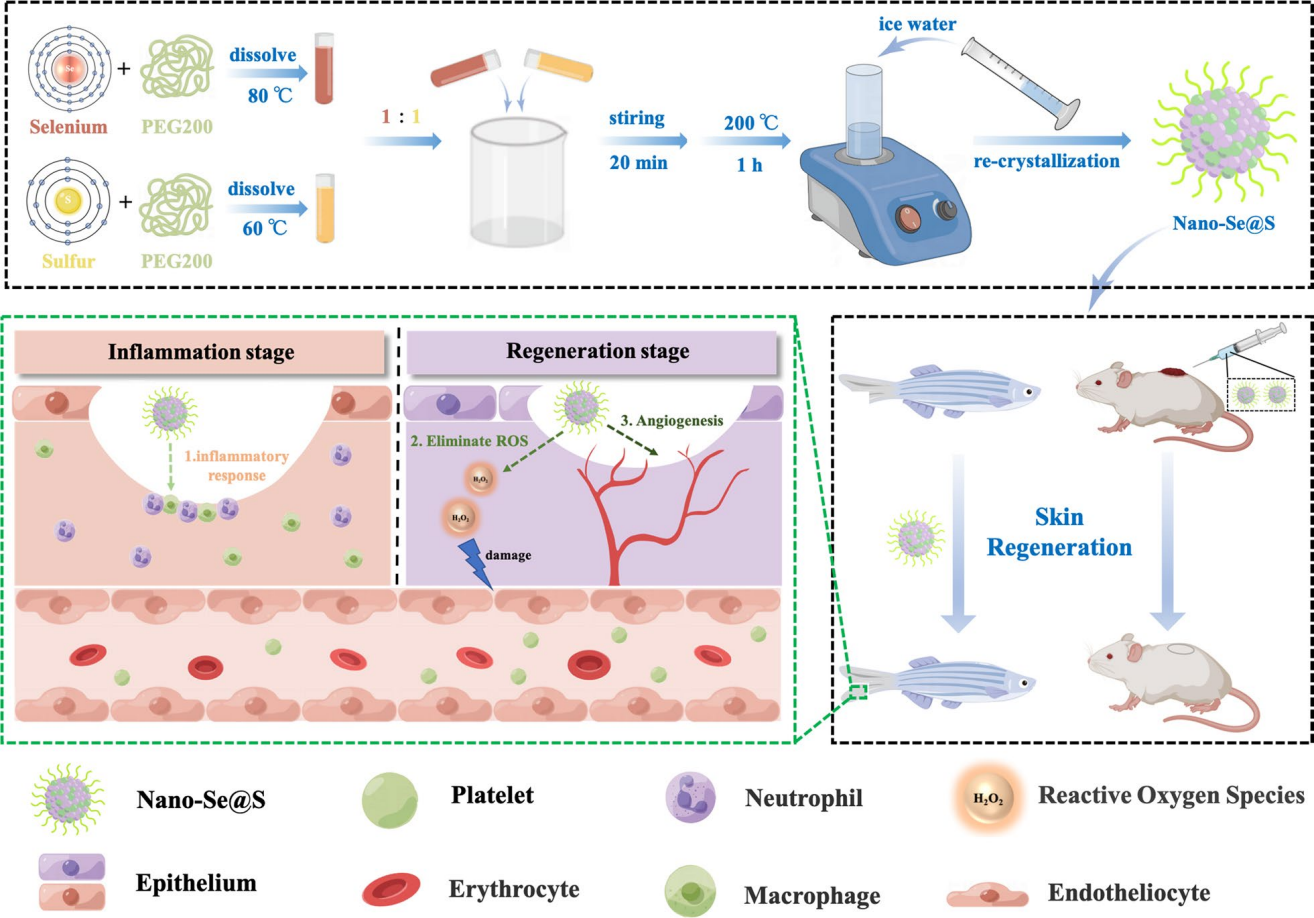

**Supplementary Information:**

The online version contains supplementary material available at 10.1186/s12951-023-01796-4.

## Background

Millions of patients suffer from regenerative deficiencies, such as refractory wound healing [1], around the world. Different therapeutic strategies for refractory wound healing have been developed in recent decades [2]. The most commonly used therapeutic strategies are growth factor dressing [3] and stem cell therapy [4]. However, alternative approaches need to be developed to accelerate tissue regeneration due to the in vivo instability of growth factors [5] and the cost of stem cell therapies [6].

Tissue regeneration is a complex physiological process that includes haemostasis [7], oxidation–reduction balance [8], inflammatory response [9], angiogenesis [10], and tissue remodeling [11]. For instance, angiogenesis delivers biological factors [12] to rebirth tissue, which is a crucial step for tissue regeneration. However, excessive reactive oxygen species (ROS) are generated around the wound site parallel to angiogenesis [13], which leads to blood vessel damage that is not beneficial for tissue regeneration [14]. At the early stage of tissue regeneration, inflammatory cells, including neutrophils and macrophages, are recruited to the site of the wound [15] to scavenge microorganisms and necrotic cells [16], providing a hospitable environment for tissue regeneration [17]. In contrast, chronic inflammation around the wound damages the proliferation and migration of tissue regeneration-related cells [18], for instance, chronic inflammation in the wounds of those with diabetes [19]. Therefore, developing new strategies with angiogenesis facilitation, ROS scavenging, and immuno-conditioning is of considerable biological and medical interest.

Selenium (Se) has a variety of biological activities, such as immunoregulation [20], redox-regulation [21], growth and development of multiple cells including but not limited to endothelial cells [22], antitumour activities [23] and reproduction [24]. These biological activities are associated with tissue regeneration. Our recent study demonstrated that hydrosoluble nanoselenium accelerated tissue regeneration through the Wnt, FGFR, and VEGFR pathways [25]. However, the cumulative toxicity of Se might be a potential limitation for its application [26]. Thus, minimizing the toxicity of Se by constructing a lower toxic chemical form or reducing the dose of Se is urgently needed. Selenium exists in inorganic, organic, and nano forms [27]; owing to the nanoform of selenium’s optimal bioavailability and lower toxicity in comparison to inorganic and organic forms [28, 29], a general strategy has emerged for lower toxic nanomaterials based on nanoselenium synthesis: employing functional molecular modifications to improve tissue/organ targeting, such as peptide modification [30] and aptamer modification [31].

Nevertheless, these methods are still limited in reducing the cumulative toxicity of selenium, and the modification method always suffers from a resource-intensive and laborious process [32]. Therefore, efficient and feasible synthesis methods for biologically active nanomaterials with lower toxicity are necessary. Sulphur (S), another oxygen family element, demonstrates excellent antibacterial activity [33] and participates in oxidative-redox regulation [34], which are both critical for tissue regeneration without toxicity [35]. Regarding this, we hypothesize that the cooperation of S with Nano-Se might be a potential strategy to reduce the toxicity of Nano-Se with reserved regeneration accelerating activity. To this end, we designed and synthesized a novel lower toxicity nanocomposite material based on selenium and sulphur for tissue regeneration.

Herein, hydrosoluble Nano-Se@S was synthesized using grey Se, sublimed sulphur, and PEG-200. Through the zebrafish tail fin regeneration model, we found that Nano-Se@S accelerates tissue regeneration with lower toxicity than Nano-Se. Transgenic zebrafish and chick embryos revealed that Nano-Se@S improved angiogenesis and ROS scavenging through transcriptomic analysis. Moreover, we found that Nano-Se@S recruits leukocytes to the wound surface at the early stage of regeneration, which contributes to sterilization during regeneration. Our study highlighted the cooperation of S as a feasible approach for synthetic Se-containing nanoparticles with reduced toxicity and broadened the application of nano selenium in regenerative medicine.

## Results and discussion

### Synthesis and characterization of Nano-Se@S

**Fig. 1:**
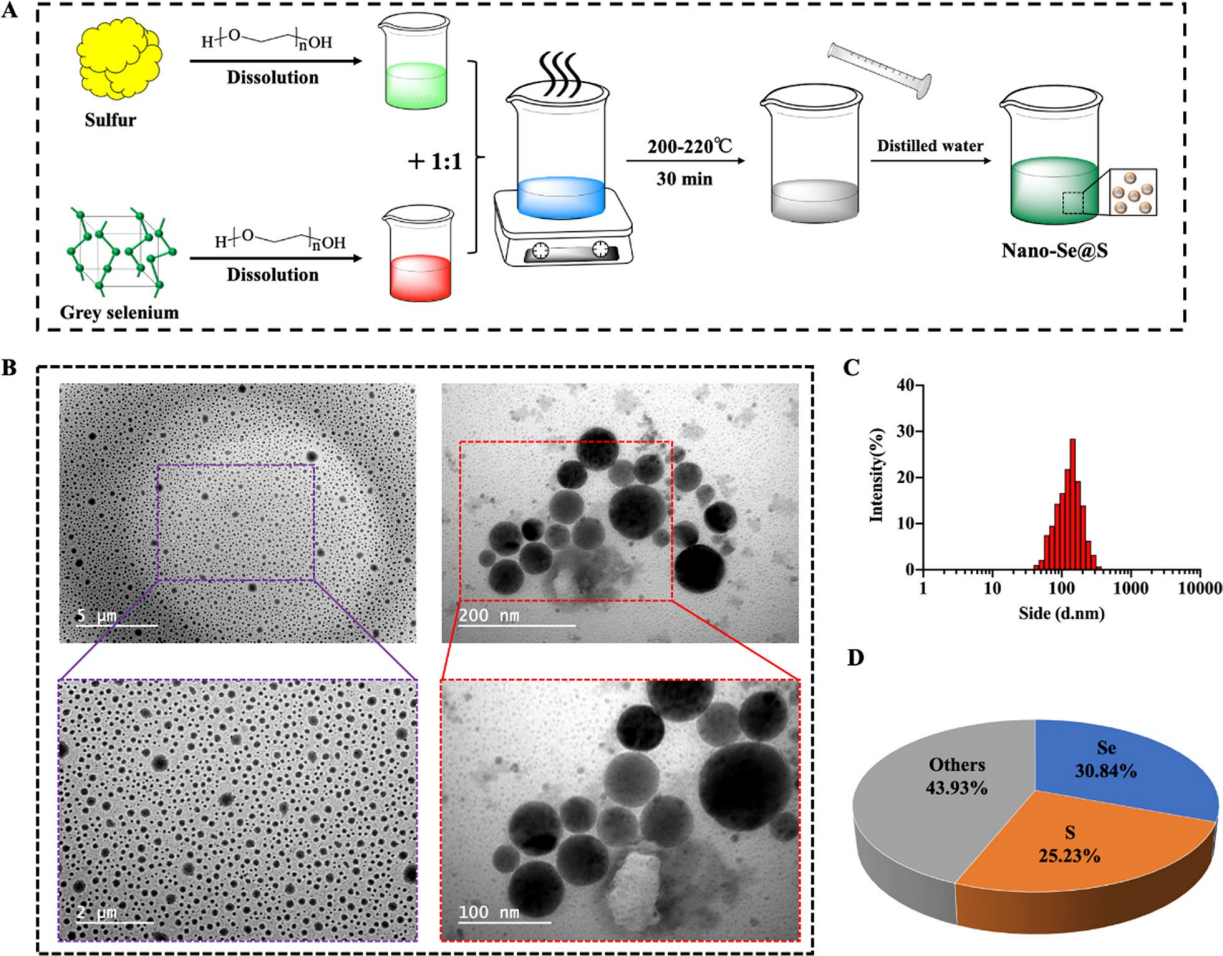
**A** Schematic illustration synthesis of Nano-Se@S. **B** Transmission electron microscope images of Nano-Se@S. **C** Particle size distribution of the Nano-Se@S. **D** Selenium and sulfur element concentrations in Nano-Se@S by ICP analysis

To synthesize selenium nanoparticles with biological activity and low toxicity, hydrosoluble Nano-Se@S was synthesized by one-pot heat methods; equal weights of grey selenium and sulphur powders were dissolved in polyethylene glycol-200 (PEG-200) and heated to 200 °C for 60 min, followed by hydration with an equal volume of cold Milli-Q water (Fig. 1A). Typical transmission electron microscopy images demonstrated that Nano-Se@S is a uniform spherical structure with a diameter of approximately 60–100 nm (Fig. 1B), which is consistent with the dynamic light scattering (DLS) results (Fig. 1C). In the synthetic Nano-Se@S, the selenium and sulphur elemental concentrations were approximately 308.4 mg/g and 253.3 mg/g, respectively, according to ICP analysis (Fig. 1D). R. Steudtner reported that selenite was converted into sulphur-selenium nanoparticles by the interaction between the toxic oxyanion selenite and the plant growth-promoting bacterium; however, this method prepared sulphur-containing selenium particles with high toxicity [36]. The lower toxicity selenium-sulphur composite synthesis method was similar to the high-temperature vacuum reaction for obtaining selenium-sulphur composites [37]. Our results indicated that a hydrosoluble spherical Nano-Se@S composite with a diameter of approximately 80 nm was successfully synthesized using the one-pot method.

### Nano-Se@S promotes tissue regeneration with reduced toxicity

**Fig. 2:**
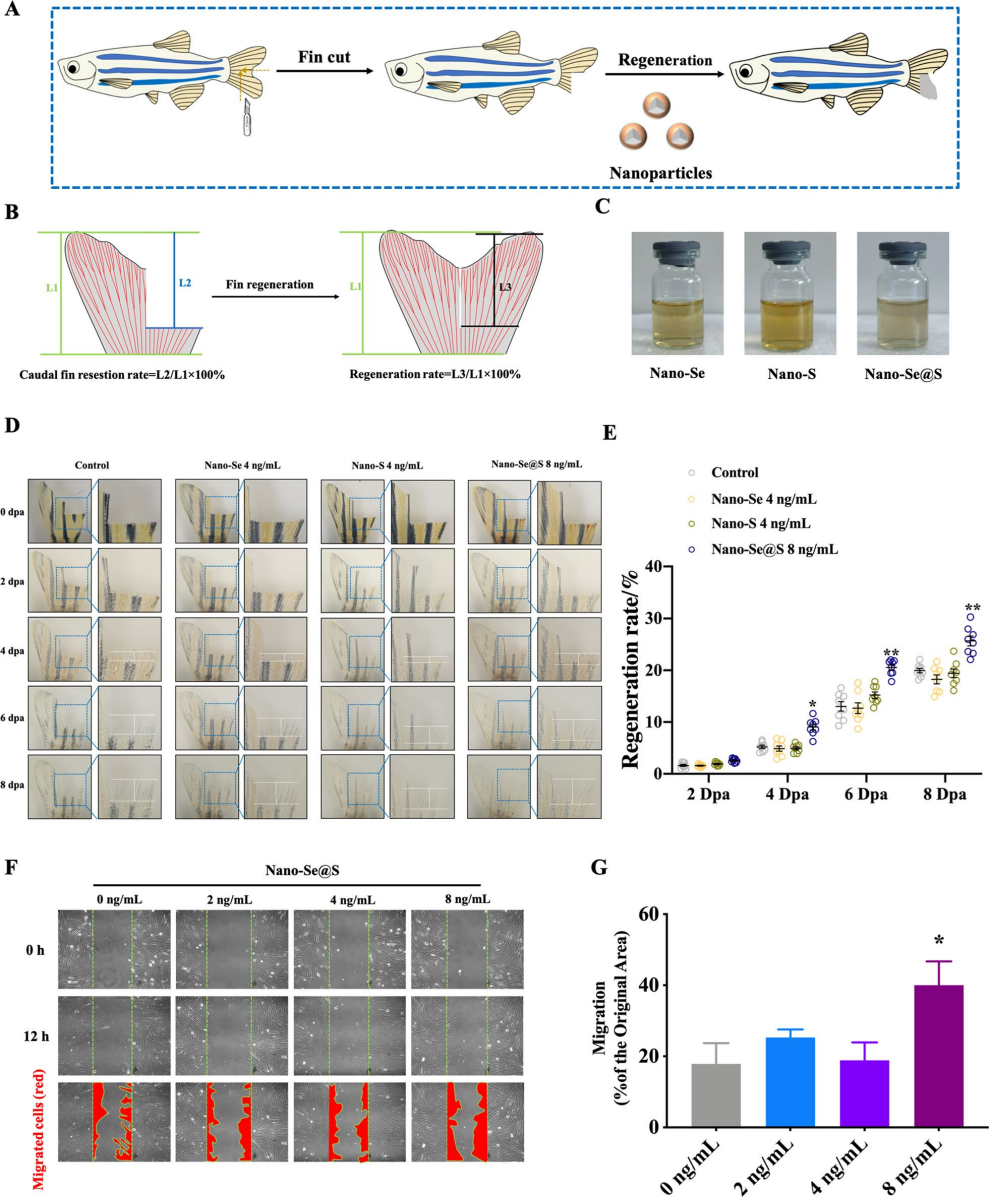
**A** Schematic illustration of Nano-Se@S accelerates tissue regeneration in zebrafish fin. **B** Schematic illustration of calculating the regeneration rate of zebrafish caudal fin. **C** Representative images of the Nano-Se, Nano-S, and Nano-Se@S solutions. **D** Representative images of the Nano-Se, Nano-S, and Nano-Se@S accelerate caudal fin regeneration. Dpa: days past amputation. **E** Regeneration rate of zebrafish caudal fin treated with Nano-Se, Nano-S, and Nano-Se@S on different days. (3 independent biological repeats n = 8). **F** Wound healing assay to evaluate the migration of HFF cells after being treated with Nano-Se@S and DMEM with 10% FBS. Cells were wounded and monitored using a microscope for 12 h. The red areas represent migrating cells. **G** The migration rate of HFF cells induced by Nano-Se-S (3 independent biological repeats n = 9). (Mean values ± SD, *P < 0.05, **P < 0.01, ***P < 0.001, ****P < 0.0001)

Recently, zebrafish have become one of the most famous experimental models [38], especially for tissue regeneration [39]. Its tail fin is well organized with vessels, nerves, and stromal structures, which comprise skin, mesenchyme, and bone frames [40, 41]. Therefore, this study employed the zebrafish tail fin regeneration model to investigate the regeneration-accelerating activities of Nano-Se@S, Nano-Se, and Nano-S. We first performed caudal fin amputation for adult zebrafish, followed by exposure to Nano-Se@S, Nano-Se, and Nano-S (Fig. 2A). The resection and regeneration rates were calculated according to the formula in Fig. 2B. A similar resection rate across all groups was used as quality control for modelling (Fig. 1S). Moreover, Nano-Se@S demonstrated significant fin regeneration accelerating activity at an 8 ng/mL concentration, which contained 4 ng/mL Nano-Se and 4 ng/mL Nano-S (Fig. 2D–E). Interestingly, exposure to 4 ng/mL Nano-Se and 4 ng/mL Nano-S demonstrated a sluggish effect on fin regeneration (Fig. 2D–E). These results indicate that Nano-Se@S achieves similar regeneration-promoting activity as Nano-Se but consumes less Se than Nano-Se through the cooperation of S. Migration of human foreskin fibroblast (HFF) cells assessed by scratch assays was performed to study whether the regeneration-accelerating activity of Nano-Se@S is conserved in mammals. Treatment with Nano-Se@S improved the migration of HFFs, as indicated by the decreased distance of wound closure (Fig. 2F–G). However, when treated with Nano-Se (4 ng/mL) and Nano-S (4 ng/mL), the migration rate of HFFs was not significantly increased (Additional file 1: Figure S4). These results collectively suggest that the incorporation of S enhances the regeneration-promoting activity of Nano-Se, which is conserved in mammals.

**Fig. 3:**
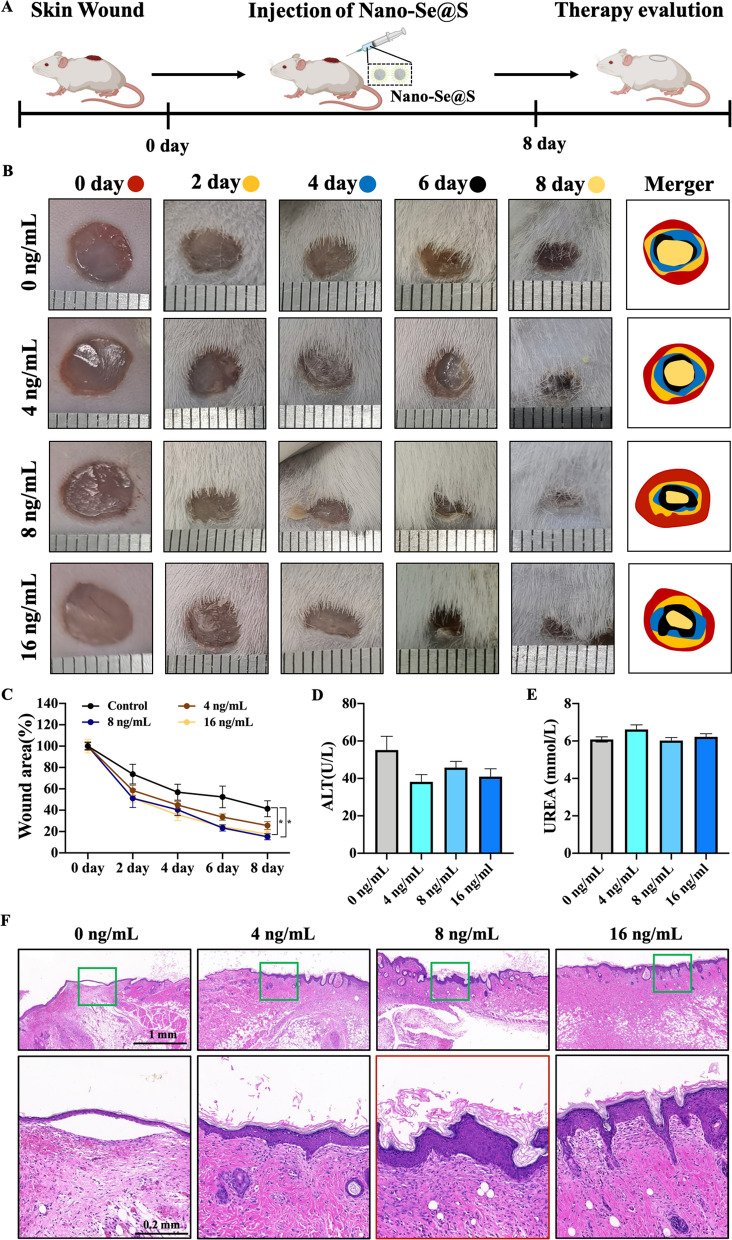
In vivo wound healing efficacy after being treated with Nano-Se@S. **A** The schematic diagram for the in vivo treatment evaluation procedure. **B** The photographs of skin wound images treated with different concentrations of Nano-Se@S. **C** Closed area ratio of skin wounds. **D** The change profiles of ALT treated with different concentrations of Nano-Se@S. **E** The change profiles of UREA treated with different concentrations of Nano-Se@S. **F** Histological graphs of skin tissue by H&E staining. (n = 5, Mean values ± SD, *P < 0.05, **P < 0.01, ***P < 0.001, ****P < 0.0001)

The effect of Nano-Se@S on tissue regeneration was also verified in mammalian mice (Fig. 3A). As shown in Fig. 3B, the skin wound healing rate was significantly accelerated. Quantification results demonstrated that the wound healing rate with 4-day exposure to Nano-Se@S (16 ng/mL) achieved a regeneration rate of 64.30% compared to that of 49.09% in the control group; this difference continued growing, and until 8 days of Nano-Se@S (16 ng/mL) exposure, the regeneration rate reached 82.42%, while the control group showed only 58.66% (Fig. 3C). After 8 days of Nano-Se@S treatment, the tissues were dissected and stained with haematoxylin and eosin (H&E) (Fig. 3F). Compared with the PBS group, regenerated epidermis was observed in the Nano-Se@S group on Day 8. Additionally, the regenerated epidermis in the Nano-Se@S group was thicker than that in the PBS group. Furthermore, a more sebaceous gland and other accessory organs were observed. Moreover, the activity of Nano-Se@S in mouse skin wound healing was significantly higher than that of Nano-Se and Nano-S (Additional file 1: Figure S6). The results of tissue sections indicated that Nano-Se@S could be used as an effective nanomaterial for the treatment of the skin wound healing process.

**Fig. 4:**
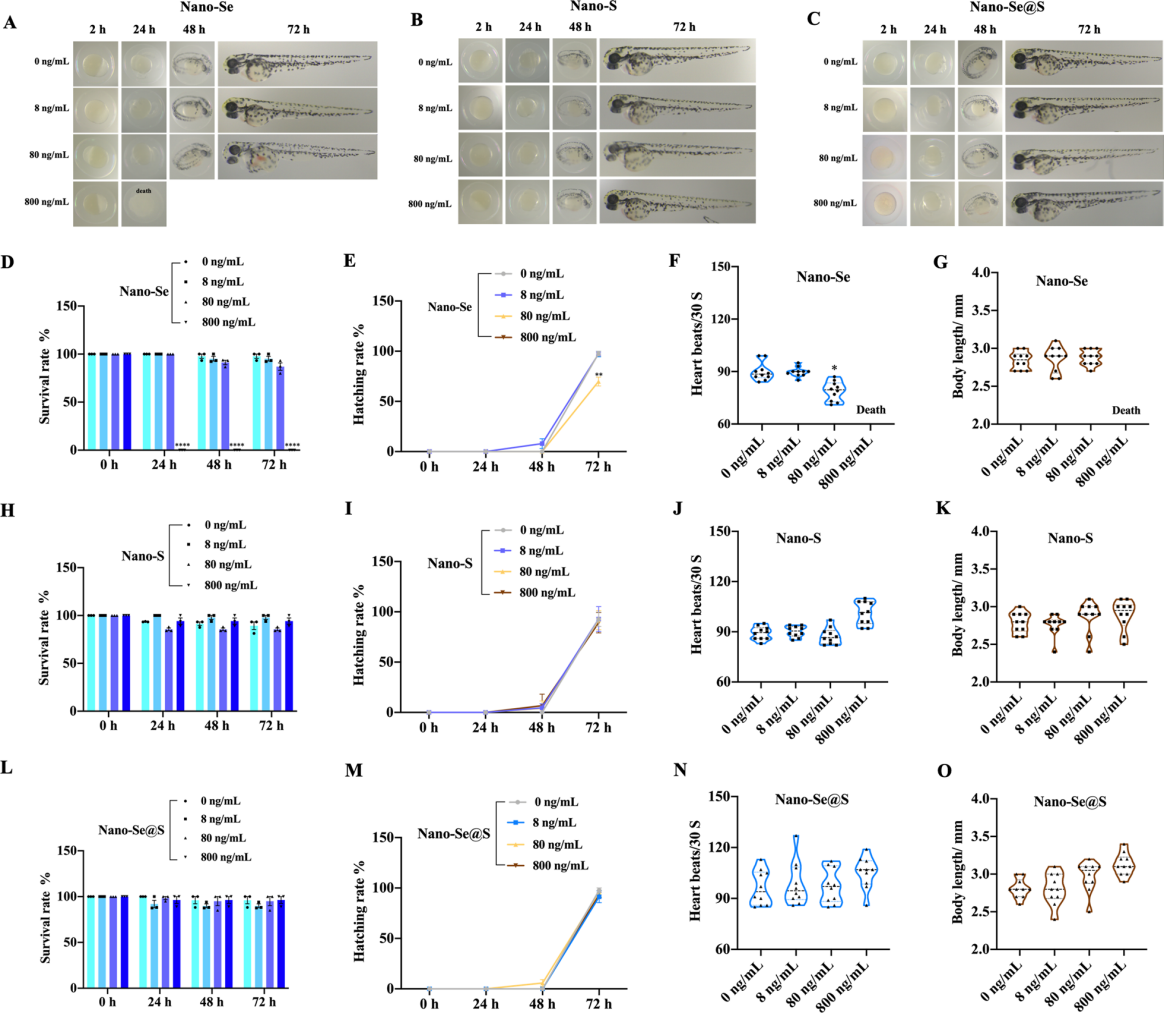
Developmental toxicity evaluation of Nano-Se, Nano-S, Nano-Se@S in zebrafish. **A** Representative images of developmental toxicity in zebrafish treated with different concentrations Nano-Se; **B** Representative images of developmental toxicity in zebrafish treated with different concentrations of Nano-S; **C** Representative images of developmental toxicity in zebrafish treated with different concentrations of Nano-Se@S; Toxicity of Nano-Se **D**, Nano-S **H**, Nano-Se@S **L** was evaluated by survival rate; Toxicity of Nano-Se **E**, Nano-S **I**, Nano-Se@S **M** was evaluated by hatching rate; Toxicity of Nano-Se **F**, Nano-S **J**, Nano-Se@S **N** was evaluated by heart beats/1 min in 72 h; Toxicity of Nano-Se **G**, Nano-S **K**, Nano-Se@S **O** was evaluated by a body length in 72 h. (n = 10, Mean values ± SD, *P < 0.05, **P < 0.01, ***P < 0.001, ****P < 0.0001)

Regarding the cumulative toxicity of selenium in vitro, the LD50 values of Nano-Se, Nano-S and Nano-Se@s in HFF were 2.428 μg/mL, 1.398 μg/mL and 5.336 μg/mL, respectively (Additional file 1: Figure S5), which indicated that the cooperation of S minimized the toxicity of Nano-Se in HFF. The in vivo toxicity of Nano-Se, Nano-S, and Nano-Se@S was assessed in zebrafish embryos and mouse major organs. As shown in Fig. 4, the toxicity of Nano-Se becomes significant as the selenium concentration increases. Excitingly, Nano-Se@S demonstrates less toxicity, as indicated by the lower decreasing range in hatching rate and heart rate and lower increasing range in lethal rate than Nano-Se at their corresponding concentrations. Nano-S shows similar toxicity to Nano-Se@S. Our previous study indicated that Nano-Se could accelerate tissue regeneration under 8 ng/mL [25], which might cause the cumulative toxicity of selenium in application to tissue regeneration. Moreover, ALT (Fig. 3D) and urea (Fig. 3E) were detected in the blood of mice, and it was proven that Nano-Se@S did not cause toxic effects in the liver and kidney of mice. However, we found obvious toxicity in the major organs (heart, liver, spleen, lung, kidney) of mice treated with different concentrations of Nano-Se, Nano-S and Nano-Se@S (Additional file 1: Figure S7). In this study, the results revealed that the cooperation of S minimized the developmental toxicity of Nano-Se. These results collectively indicate that tissue regeneration-stimulated Nano-Se@S activity is conserved between zebrafish and mice with lower development toxicity than Nano-Se.

### Transcriptome analysis of Nano-Se@S on zebrafish tail fin regeneration

**Fig. 5:**
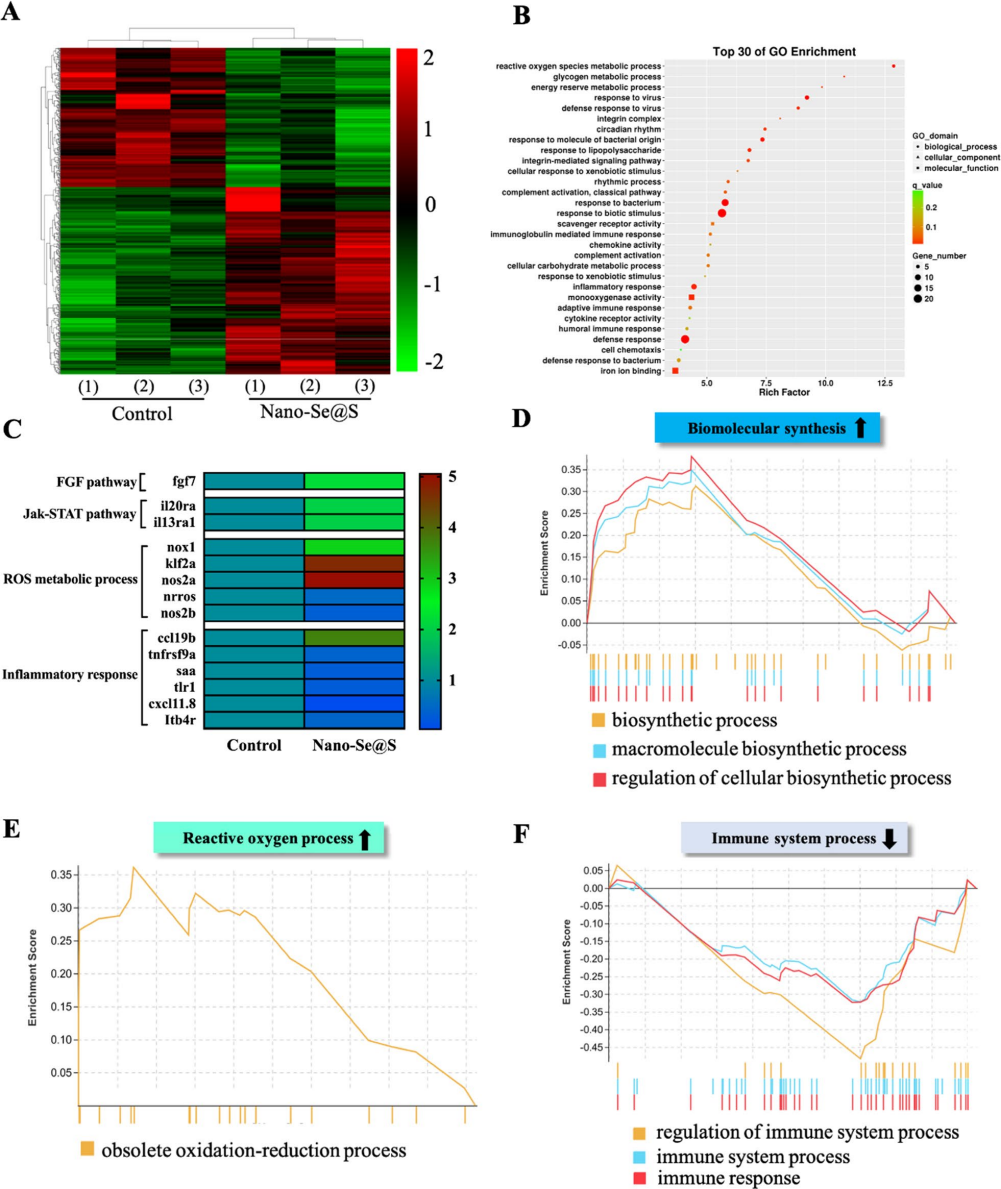
RNA-Seq analysis results of Nano-Se@S in zebrafish regeneration fin. **A** Heat maps of significantly upregulated and downregulated genes (fold change ≥ two and P < 0.05). **B** Gene Ontology (GO) enrichment analysis of the identified differentially expressed genes. The 30 most significantly enriched GO are shown. **C** Heat maps of significantly upregulated and downregulated genes related to FGF, Jak-STAT pathway, ROS metabolic process, and inflammatory response. **D** GSEA analysis of biomolecular synthesis. **E** GSEA analysis of reactive oxygen process. **F** GSEA analysis of immune system process

To explore the potential mechanisms by which Nano-Se@S accelerates tissue regeneration, transcriptomic analysis was performed. Significant differences in transcripts and some characteristics between the Nano-Se@S and control groups were indicated using principal component analysis (PCA) (Additional file 1: Figure S2A). Moreover, heatmap analysis (Fig. 5A) and volcano plots (Additional file 1: Figure S2B) demonstrated that 218 genes were upregulated; in comparison, 295 genes were downregulated after treatment with Nano-Se@S. Our previous transcriptomic analysis of zebrafish tail fins indicated that 400 genes were upregulated. In contrast, 40 genes were downregulated during treatment with Nano-Se [25], which illustrated that the cooperation of S alters the gene expression profile of Nano-Se in tissue regeneration. Gene Ontology enrichment analysis indicated that the reactive oxygen species (ROS) metabolic process and inflammatory response contributed to these upregulated genes (Fig. 5B). To accurately analyse the influence of differentially expressed genes on Nano-Se@S in tissue regeneration. The GSEA results further strengthened this, as indicated by the activation of the oxidation‒reduction process (Fig. 5E) and biomolecular synthesis-related processes in vivo (Fig. 5D) and the subsiding immune system process (Fig. 5F). Compared with previous Nano-Se transcriptome analysis [25], Nano-Se@S is more involved in regulating immune function than Nano-Se. In addition, Nano-Se@S (Fig. 5C) and Nano-Se jointly regulate the FGF signalling pathway, which is conducive to promoting cell proliferation and migration during tissue regeneration [42]. These results suggest that Nano-Se@S may reduce the inflammatory reaction and promote biosynthesis to facilitate tissue regeneration.

### Nano-Se@S protects blood vessels through ROS scavenging

**Fig. 6:**
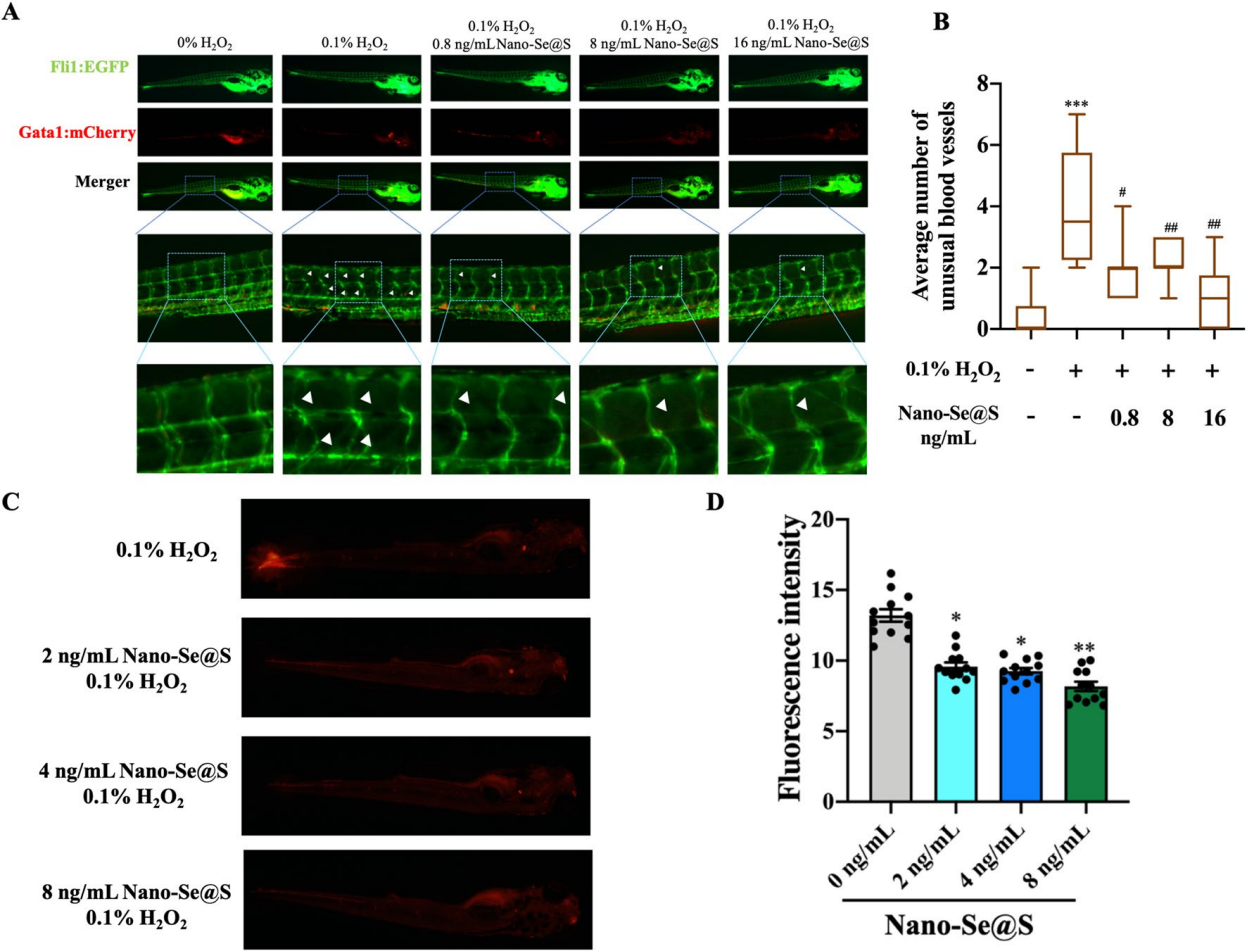
**A** Nano-Se@S protects against H_2_O_2_-induced vascular damage in transgenosis zebrafish (*fli1*:EGFP/*gata1*:mCherry) which blood vessels were labeled with green fluorescence and blood with red fluorescence. **B** Quantitative average of number of unusual vessels treated with Nano-Se@S in in transgenosis zebrafish (*fli1*:EGFP *fli1*:EGFP/*gata1*:mCherry) (n = 8). **C** DHE probe detected the inhibition of reactive oxygen species in zebrafish by Nano-Se@S. **D** Quantitative fluorescence analysis of reactive oxygen species in zebrafish (n = 12). (Mean values ± SD, *: Significant difference compared with control group, *P < 0.05, **P < 0.01, ***P < 0.001, ****P < 0.0001, #: Significant difference between 0.1% H_2_O_2_ and 0.1% H_2_O_2_ + Nano-Se@S group, #P < 0.05, ##P < 0.01, ###P < 0.001, ####P < 0.0001)

According to the RNA-Seq results, the ROS metabolic process was significantly affected by Nano-Se@S. ROS play different roles at different stages of tissue regeneration in wound sites [43, 44]; they can upregulate regeneration-related cell proliferation and migration at the late stage of tissue regeneration [43]. More importantly, this will lead to vascular damage [45] during angiogenesis in tissue regeneration. To study whether Nano-Se@S has a protective effect on vascular injury caused by ROS, a double transgenic zebrafish (*fli1*: EGFP/*gata1*: mCherry), in which endothelial cells and red blood cells are labelled with a green and red fluorescent protein, was employed. When double transgenic zebrafish were treated with 0.1% H_2_O_2_ for 20 min, apparent blood vessel damage, such as narrowing and malformation in the torso of zebrafish, was observed (Fig. 6A), which indicated that excessive ROS could impact the normal function of blood vessels and that Nano-Se@S could significantly reduce the vascular injury caused by excessive ROS in zebrafish (Fig. 6A, B).

Moreover, DHE (ROS fluorescent probe) was employed to evaluate the function of scavenging ROS by Nano-Se@S. Figure 6C and D show that the superoxide indicated by red fluorescence significantly decreased in zebrafish treated with Nano-Se@S via a dose-dependent effect.

### Nano-Se@S promotes angiogenesis

**Fig. 7:**
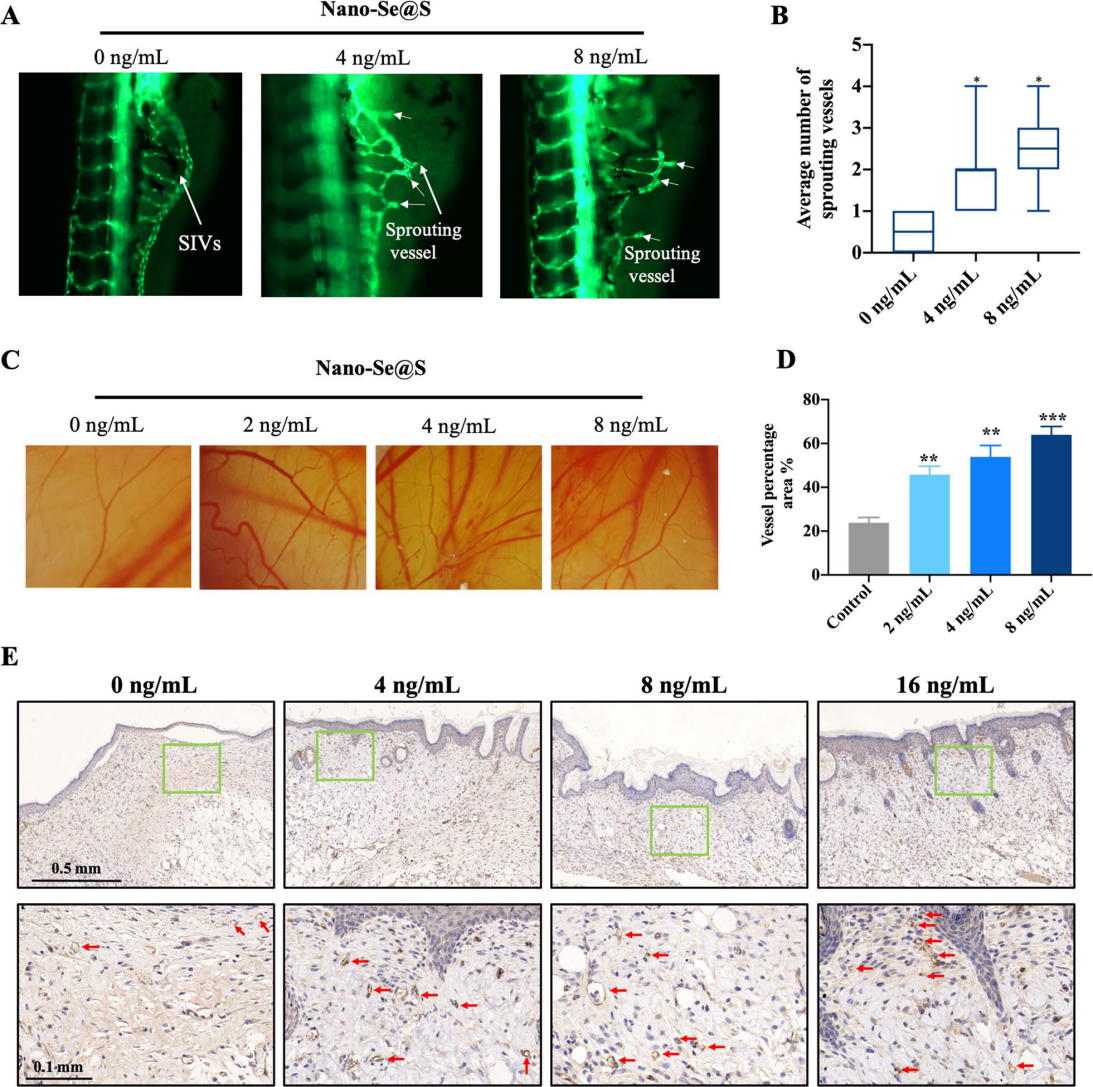
Angiogenesis promoted by Nano-Se@S. The 24 hpf embryos were treated with different concentrations of Nano-Se-S for 48 h. **A** Representative fluorescence microscopy images in transgenesis zebrafish (*fli1*:EGFP) showed blood vessels labeled with green fluorescence. **B** The quantitative average number of sprouting vessels treated with Nano-Se@S in transgenic zebrafish (*fli1*:EGFP) (n = 8). **C** Nano-Se@S promotes angiogenesis of the allantoic membrane of chicken embryos. **D** Quantitative vessel percentage area treated with Nano-Se@S in the chicken embryo (n = 5). **E** CD31 immunohistochemistry (IHC) shows vascularization within the Nano-Se@S granulation tissue. Arrow indicate angiogenesis (n = 5) (Mean values ± SD, *P < 0.05, **P < 0.01, ***P < 0.001, ****P < 0.0001)

Angiogenesis is crucial for tissue regeneration [46]; according to the results, Nano-Se@S could protect against vascular damage due to ROS. We detected that Nano-Se@S promotes blood vessel regeneration in a zebrafish angiogenesis model in the subintestinal vein (SIVs), and 24-h postfertilization (hpf) zebrafish embryos were incubated with Nano-Se@S for 48 h. The number of SIVs was also significantly increased after treatment with Nano-Se@S (Fig. 7A, B). Moreover, the number of branches of blood vessels increased significantly after Nano-Se@S treatment in our chicken embryo allantoic membrane experiment (Fig. 7C), and the vessel percentage area improved considerably with Nano-Se@S (Fig. 7D), indicating the stimulating effect of Nano-Se@S on angiogenesis. Moreover, vascular density, visualized through immunohistochemistry (IHC) for cluster of differentiation 31 (CD31) in mouse regeneration skin, was also positively correlated with Nano-Se@S (Fig. 7E). These results are consistent with our previous results that Nano-Se promotes angiogenesis through the VEGFR signalling pathway [25], indicating that the biological effect of selenium on angiogenesis is still maintained in the Se-S nanocomposite.

### Nano-Se@S initiates the accumulation of inflammatory cells after wound healing

**Fig. 8:**
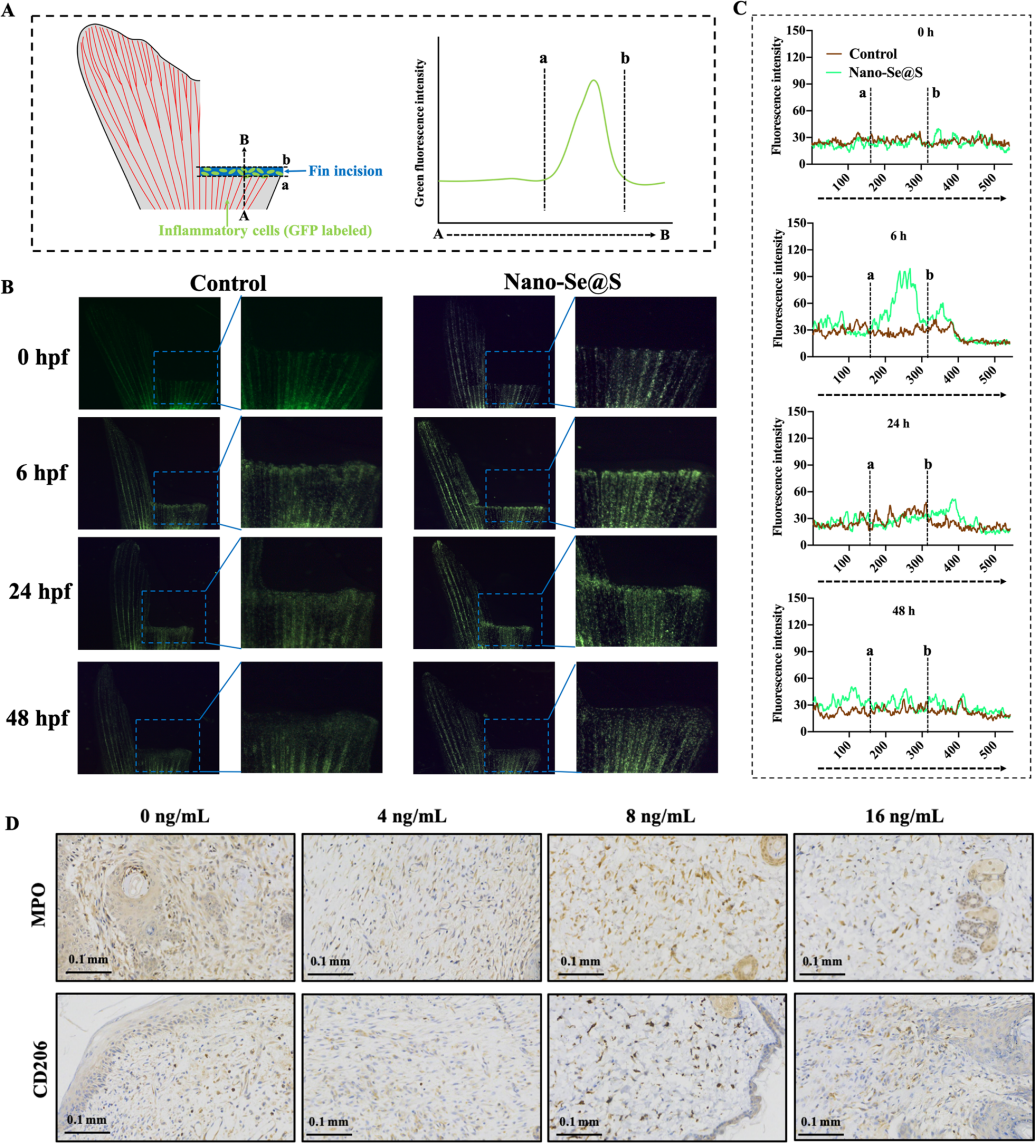
The inflammatory response is promoted by Nano-Se@S (8 ng/mL). **A** Schematic illustration of testing the aggregates of inflammatory cells at the wound site in zebrafish fin. **B** Representative fluorescence microscopy images in transgenic zebrafish (*coro1a*:EGFP) in which inflammatory cells were labeled with green fluorescence. **C** Quantitative fluorescence analysis of inflammatory cells at the wound site in zebrafish fin treated with Nano-Se@S in transgenic zebrafish (*coro1a*:EGFP). **D** Representative MPO (neutrophils) and CD206 (M2 macrophages) IHC images of regeneration skin tissue treated with different concentration of Nano-Se@S. Scale bars, 100 μm

In addition to blood vessels, inflammatory cells are crucial for tissue regeneration during different periods [47]. According to our RNA-Seq results, the inflammatory response contributed to the enrichment of these upregulated genes (Fig. 5C). To investigate whether Nano-Se@S has regulatory activity on inflammatory cells, we used transgenic zebrafish (*coro1a*:EGFP) in which inflammatory cells (neutrophils and macrophages) were labelled with green protein to evaluate the effect of Nano-Se@S on wound inflammatory cells during early-stage tissue regeneration (Fig. 8A). As shown in Fig. 8B and C, 6 h after fin damage, we found that the Nano-Se@S group could significantly increase inflammatory cells gathered at the wound site compared with the control group. Subsequently, the aggregation of inflammatory cells in the wound gradually decreased until 48 h, and the inflammatory response returned to 0 hpf. These results indicated that Nano-Se@S improved inflammatory cell accumulation at the wound site during the early tissue regeneration stage. Moreover, after treatment with Nano-Se@S for 8 days, CD206^+^ macrophages increased and myeloperoxidase-positive (MPO^+^) neutrophils decreased (Fig. 8D) in the regeneration skin tissue around the wound, which was conducive to tissue regeneration. We found that compared to Nano-Se [25], Nano-Se@S regulates inflammatory cells collected at the wound site during early tissue regeneration, which is a new mechanism to accelerate tissue regeneration that may be attributed to sulphur.

Recent studies have shown that ROS accumulated in the wound not only induce strong inflammatory reactions to make wounds vulnerable [48] but also inhibit the functions of endogenous stem cells and macrophages to hinder wound tissue regeneration simultaneously [49]. In addition to ROS produced by the wound itself, ROS produced by bacterial infection can also cause serious damage to blood vessels and endothelial cells, resulting in chronic wound formation [50, 51]. In this nanocomposite, selenium is known to scavenge reactive oxygen species and regulate inflammation [52], while sulphur has strong antibacterial activity [53]. Selenium probably acts as a cleaner at the site of bacterial infection, thereby reducing reactive oxygen species produced by bacteria.

In addition to oxidative metabolism and inflammation, various signalling pathways are involved in tissue regeneration. In this study, according to RNA-seq results, we found that FGF (*fgf7*) and the Jak-STAT signalling pathway (*il20ra, il13ra1*) were upregulated after treatment with Nano-Se@S-induced tissue regeneration. The importance of these two signalling pathways has been stressed in zebrafish and mouse studies [54, 55]. Thus, our results are consistent with those of previous investigations.

Taken together, we highlight Nano-Se@S as an accelerator for tissue regeneration with lower toxicity than Nano-Se. Mechanistically, Nano-Se@S protects against vascular damage by scavenging ROS activity and promotes angiogenesis, which is one of the classical mechanisms during tissue regeneration. Moreover, regulating the inflammatory response is beneficial to wound healing.

## Conclusion

In summary, we illustrated one-pot methods for hydrosoluble Nano-Se@S synthesis and revealed that Nano-Se@S facilitates tissue regeneration with reduced toxicity using mice, zebrafish, chick embryos, and human cells. Mechanistically, Nano-Se@S protects blood vessels by scavenging ROS and promoting angiogenesis. Moreover, Nano-Se@S moderates the immune microenvironment around the wound. This work demonstrates the possibility of applying Nano-Se@S for skin regeneration. Compared to clinical methods (debridement, antibacterial and negative pressure closed drainage), this nanocomposite targets the microenvironment of wound healing, including inflammation, angiogenesis and cell migration, and may improve the compliance and reduce the discomfort of clinical patients, providing a safe and effective treatment for wound healing in patients. Further in-depth research on the appropriate pharmaceutical formulations and antibacterial effects of Nano-Se@S to provide a foundation for human refractory wound clinical therapy will be performed in future studies.

## Materials and methods

### Materials

Selenium (Se), sulphur (S), MS-222, and dihydroethidium were purchased from Sigma–Aldrich Chemical Co., and PEG200 was purchased from Sangon Biotech Co., Ltd. without further purification. DMEM was purchased from Gibco Co.,Ltd. FBS was from Biological Industries Co., Ltd. Ultrapure Milli-Q water was used in all experiments.

### Cell lines

Cell lines, including human skin fibroblast cells (HFFs), were purchased from American Type Culture Collection (ATCC, Manassas, VA, USA).

### Zebrafish transgenic lines and zebrafish maintenance

The transgenic line (*fli1*: *EGFP/gata1*: mCherry), where blood vessels were labelled with green fluorescence and blood was marked in red, was used to observe fin blood vessels. The transgenic line (*coro1a*: EGFP) in which inflammatory cells were labelled with green fluorescence was used to maintain the inflammatory response during early regeneration. Adult zebrafish were maintained in a water circulation system; the water temperature was held at 28 °C, the conductivity was adjusted to 500–550, and the pH of the culture water was adjusted to 7.5–8.0. The larval zebrafish were placed in a constant temperature and light incubator; the temperature was maintained at 28 °C, and the spare time was 14 h/day.

### Synthesis of Nano-Se@S

Nano-Se@S was synthesized by dissolving 10 mg of grey Se in 10 mL of PEG200 solution under magnetic stirring for 10 min at 80 °C when the solution turned from colourless to dark brown, and 10 mg of sulphur powder was dissolved in 10 mL of PEG200 solution under magnetic stirring for 10 min at 60 °C when the solution turned yellow. Next, the dark brown and yellow solutions were mixed with a constant volume and incubated at 200 °C for 60 min to observe that the solution became brown and that the grey selenium and sulphur were completely dissolved. Then, an equal volume of water (4 °C) was added to the reaction solution. Finally, the answer was filtered with a 0.22 µm filter membrane to obtain the Nano-Se@S solution.

### Characterization of Nano-Se@S

Transmission electron microscopy (TEM) samples were prepared by dispersing the pieces onto a holey carbon film on copper grids. The micrographs were obtained on a Tecnai G220 (Shimadzu, Japan) at 200 keV. A dynamic light scattering (DLS) particle size analyser (Malvern 2000, USA) was used to determine the hydrophilic diameters of the particles. The concentration of Se was detected by inductively coupled plasma-atomic emission spectrometry (ICP‒AES) (Thermo Scientific, iCAP 7400, USA). All measurements were performed at room temperature unless otherwise mentioned.

### Zebrafish fin regeneration model

Wild-type adult zebrafish and transgenic adult zebrafish (5 months old) were anaesthetized in 0.1% MS-222, and caudal fins were amputated using a scalpel. Then, the experimental group was treated with Nano-Se@S every other day for 8 days. Finally, zebrafish were anaesthetized with 0.1% MS-222, and fin regeneration length was determined using fluorescence microscopy (Mingmei Optoelectronics Technology Co. Ltd.) for analysis at different time points. The regeneration rate of zebrafish caudal fins treated with Nano-Se, Nano-S and Nano-Se@S. All animal experiments complied with the Arrive guidelines and were carried out by the National Institutes of Health Guide for the Care and Use of Laboratory Animals.

### Mouse skin wound healing model

Mice were provided by Guangdong Medical Laboratory Animal Center. Adult mice were anaesthetized with pentobarbital sodium. After the mice were anaesthetized, a skin perforator with a diameter of 6 mm was used to construct a skin wound healing model on the backs of the mice. After the wound healing model was constructed, different concentrations of Nano-Se@S were administered subcutaneously at the two specific spot sites around the wound (dose: 2.5 mL/kg). Acquired images and ImageJ were used to quantify the wound healing rate based on five different fields of vision. Regeneration skin tissue was additionally collected from these mice for CD31, MPO, CD206 and H&E staining, while the major organs (heart, liver, spleen, lung and kidney) were used for H&E staining for toxicity research. The animals were maintained in accordance with the Guide for the Care and Use of Laboratory Animals issued by the National Institutes of Health and approved by the Laboratory Animal Ethics Committee of Jinan University.

### Extraction and purification of total RNA from zebrafish-regenerated fin tissue and bioinformatics analysis

After tail cutting 30 wild-type (WT) zebrafish, they were randomly divided into two groups: one group was cultured in system aquaculture (control group, n = 15), and the other group was treated with Nano-Se@S (8 ng/mL) every other day for 10 days. Then, the regenerated fins were collected. Total RNA was obtained with an RNeasy Micro Kit according to standard operating procedures. After electrophoresis, an RNAClean XP Kit and RNase-Free DNase Set were used to purify total RNA. Only high-quality RNA samples (OD260/280 = 1.8–2.0, RIN  ≥ 9.5, 28S:18S = 1.6–2.4) were used to construct a sequencing library, Illumina HiSeq X10 was used, and RNA purification was performed at Shanghai Bohao Biotechnology Co., Ltd. Reverse transcription, library construction, and sequencing were performed. For bioinformatics analysis, the expression level of each transcript was calculated based on the number of fragments per million exons per million mapped reads (FPKM) method. RSEM was used to quantify gene abundance. The R statistical software package DESeq2 (fold change ≥ 2 and P value < 0.05) was used to identify differentially expressed genes with a false discovery rate (FDR) cut-off value < 0.05.

### Construction of a vascular injury model induced by ROS

The double transgenic line (*fli1*: *EGFP/gata1*: mCherry) larvae zebrafish were placed in 96-well plates (1/well), and cultured water was used to prepare 0.1% H_2_O_2_ at 100 μL in the model group and 0.1% H_2_O_2_ at 100 μL in total volume and different concentrations of Nano-Se@S in the administration group. After 20 min, all groups were photographed under a fluorescence microscope to observe the blood vessels.

### ROS were detected by DHE in zebrafish

Wild-type larval zebrafish were placed in 96-well plates (1/well), and cultured water was used to prepare 100 μL of 0.1% H_2_O_2_ in the model group. Then, 100 μL of 0.1% H_2_O_2_ in total volume and different concentrations of Nano-Se@S were added to the administration group for 20 min. Then, the liquid was removed, and DHE solution (50 μL/well) was added to the stain for 10 min. Finally, the red fluorescence in the zebrafish was observed under a fluorescence microscope, and quantitative fluorescence analysis was performed by ImageJ. These steps were shielded from light.

### Detection of inflammatory cells at the wound site in zebrafish fins

Transgenic (*coro1a*: EGFP) adult zebrafish (5 months old) were anaesthetized in 0.1% MS-222, and caudal fins were amputated using a scalpel. Then, the experimental group was treated with Nano-Se@S every day for 48 h. Finally, zebrafish were anaesthetized with 0.1% MS-222, and fin wound site inflammatory cells were collected by fluorescence microscopy (Mingmei Optoelectronics Technology Co. Ltd.) for analysis at different time points, and ImageJ was used to analyse the green fluorescence intensity of caudal fin wounds of the zebrafish at other time points.

### Scratch test assay

The digested cells were inoculated at a dilution of 4 × 10^4^ cells/well into a six-well plate and incubated in a 37 °C 5% CO_2_ incubator for 24 h to form a monolayer treated with DMEM (0.5% FBS) for 12 h for starvation. A line was then scratched on the culture using a tip (200 mL pipet) orthogonal to the mark on the plate, and the six-well plate was shaken carefully with PBS to remove floating or dead cells. Nano-Se, Nano-S and Nano-Se@S were diluted with 0.5% starvation medium to different concentrations, and the same volume was added to each well and cultured for 24 h. Images were acquired with a microscope, and ImageJ was used to quantify cell migration activity. The average migration rate was calculated based on five different fields of vision (3 independent biological repeats with a total n = 9).

### In vitro cytotoxicity of Nano-Se

The HFF cells were inoculated at a dilution of 2 × 10^3^ cells/well into a 96-well plate and incubated in a 37 °C 5% CO_2_ incubator for 24 h. Then, the cells were treated with DMEM (0.5% FBS) for 24 h for starvation, and the medium was removed. Nano-Se, Nano-S and Nano-Se@S were diluted with 0.5% starvation medium to different concentrations, and the same volume was added to each well. The cells were cultured for 48 h, and the medium was removed. Next, 100 μL/well CKK-8 solution was added for 1 h. Finally, the absorbance of each well at 450 nm was measured using a microplate analyser. Finally, PRISM software was used to calculate the LD50 of nano-Se with three replicates in each group.

### Evaluation of the effects of Nano-Se, Nano-S, and Nano-Se@S on the developmental toxicity of zebrafish

Wild-type juvenile zebrafish (2 h after fertilization) were plated into 24-well plates, and each well contained ten strips. Then, Nano-Se, Nano-S, and Nano-Se@S were treated with 0, 8, 80, and 800 ng/mL every day for 72 h. The number of zebrafish that survived and hatched in each well was recorded every 24 h until 72 g. The heartbeat of each zebrafish in each group was counted (times/min), and the zebrafish body length in each group was measured at 72 g.

### Statistical analysis

Data are expressed as the mean ± standard deviation (SD). Data were analysed using GraphPad Prism 8.0 software (one-way ANOVA or two-way ANOVA, (*) for p < 0.05, (**) for p < 0.01, (***) for p < 0.001, and (****) for p < 0.0001).

## Supplementary Information

**Additional file 1: Figure S1.** Caudal fin resection rate of zebrafish treated with Nano-Se, Nano-S, Nano-Se@S. **Figure S2. (A)** Principal component analysis (PCA) was performed based on differentially expressed genes from regenerated tail fins of two groups. Each data point corresponds to the PCA analysis of each sample. **(B)** Volcano plots show the identified upregulated and downregulated genes by Nano-Se@S. **(C)** KEGG pathway enrichment analysis of the identified differentially expressed genes. The 30 most significantly enriched pathways are shown. **Figure S3. (A)** Detection of AST index in mouse blood; **(B)** Detection of AST index in mouse blood. **Figure S4. (A)** Wound healing assay to evaluate the migration of HFF cells after being treated with Nano-Se and DMEM with 10% FBS. Cells were wounded and monitored using a microscope for 12 h. The red areas represent migrating cells. (**B)** The migration rate of HFF cells induced by Nano-Se. **(C)** Wound healing assay to evaluate the migration of HFF cells after being treated with Nano-S and DMEM with 10% FBS. Cells were wounded and monitored using a microscope for 12 h. The red areas represent migrating cells. (**D)** The migration rate of HFF cells induced by Nano-S (3 independent biological repeats n = 9). **Figure S5.** (A) The LD50 of the Nano-Se (0, 0.625, 1.25, 2.5, 5 μg/mL) in HFF cells by CCK8 assay; (B) The LD50 of the Nano-S (0, 0.625, 1.25, 2.5, 5 μg/mL) in HFF cells by CCK8 assay; (C) The LD50 of the Nano-S (0, 0.625, 1.25, 2.5, 5,10 μg/mL) in HFF cells by CCK8 assay. (n = 3). **Figure S6. (A)** The photographs of skin wound images treated with Nano-Se (4 ng/mL), Nano-S (4 ng/mL), Nano-Se@S (8 ng/mL). **(B)** Closed area ratio of skin wounds. (n = 6, Mean values ± SD, *P < 0.05, **P < 0.01, ***P < 0.001, ****P < 0.0001). **Figure S7.** H&E staining of major organs (heart, liver, spleen, lung and kidney) showing the biosafety of different drug formulations, Scale bar is 300 μm. Yellow box: 5 times magnification image.

## Data Availability

The data that support the findings of this study are available from the corresponding author upon reasonable request.

## Abbreviations

PEG: Polyethylene glycol
DLS: Dynamic light scattering
TEM: Transmission electron microscopy
ICP: Inductively coupled plasma
HFF: Human skin fibroblast
DHE: Dihydroethidium
ROS: Reactive oxygen species
hpf: Hours post fertilization
dpa: Days post amputation
