# Supplementary material for: Turning gray selenium and sublimed sulfur into a nanocomposite to accelerate tissue regeneration by isothermal recrystallization
Source: J Nanobiotechnology. 2023 Feb 21;21:57. doi: 10.1186/s12951-023-01796-4 (PMC9942369; doi:10.1186/s12951-023-01796-4)
Supplement: Supplementary file 1 — Additional file 1: Figure S1. Caudal fin resection rate of zebrafish treated with Nano-Se, Nano-S, Nano-Se@S. Figure S2. (A) Principal component analysis (PCA) was performed based on differentially expressed genes from regenerated tail fins of two groups. Each data point corresponds to the PCA analysis of each sample. (B) Volcano plots show the identified upregulated and downregulated genes by Nano-Se@S. (C) KEGG pathway enrichment analysis of the identified differentially expressed genes. The 30 most significantly enriched pathways are shown. Figure S3. (A) Detection of AST index in mouse blood; (B) Detection of AST index in mouse blood. Figure S4. (A) Wound healing assay to evaluate the migration of HFF cells after being treated with Nano-Se and DMEM with 10% FBS. Cells were wounded and monitored using a microscope for 12 h. The red areas represent migrating cells. (B) The migration rate of HFF cells induced by Nano-Se. (C) Wound healing assay to evaluate the migration of HFF cells after being treated with Nano-S and DMEM with 10% FBS. Cells were wounded and monitored using a microscope for 12 h. The red areas represent migrating cells. (D) The migration rate of HFF cells induced by Nano-S (3 independent biological repeats n = 9). Figure S5. (A) The LD50 of the Nano-Se (0, 0.625, 1.25, 2.5, 5 μg/mL) in HFF cells by CCK8 assay; (B) The LD50 of the Nano-S (0, 0.625, 1.25, 2.5, 5 μg/mL) in HFF cells by CCK8 assay; (C) The LD50 of the Nano-S (0, 0.625, 1.25, 2.5, 5,10 μg/mL) in HFF cells by CCK8 assay. (n = 3). Figure S6. (A) The photographs of skin wound images treated with Nano-Se (4 ng/mL), Nano-S (4 ng/mL), Nano-Se@S (8 ng/mL). (B) Closed area ratio of skin wounds. (n = 6, Mean values ± SD, *P < 0.05, **P < 0.01, ***P < 0.001, ****P < 0.0001). Figure S7. H&E staining of major organs (heart, liver, spleen, lung and kidney) showing the biosafety of different drug formulations, Scale bar is 300 μm. Yellow box: 5 times magnification image. [file 12951_2023_1796_MOESM1_ESM.docx]

**Additional file**

**Turning gray selenium and sublimed sulfur into a nanocomposite to accelerate tissue regeneration by isothermal recrystallization**

Jieqiong Cao^1,2#^, Yibo Zhang^1#^, Yiqi Yang^1,2#^, Junye Xie^1^, Zijian Su^1^, Fu li^1^, Jingsheng Li^1^, Zhang Bihui^1^, Zhenyu Wang^1^, Peiguang Zhang^1^, Zhixin Li^1^, Liu He^1^, Hongwei Liu^2^, Wenjie Zheng^3^, Shuixing Zhang^2^*, An Hong^1,2^*, Xiaojia Chen^1,2^*

**
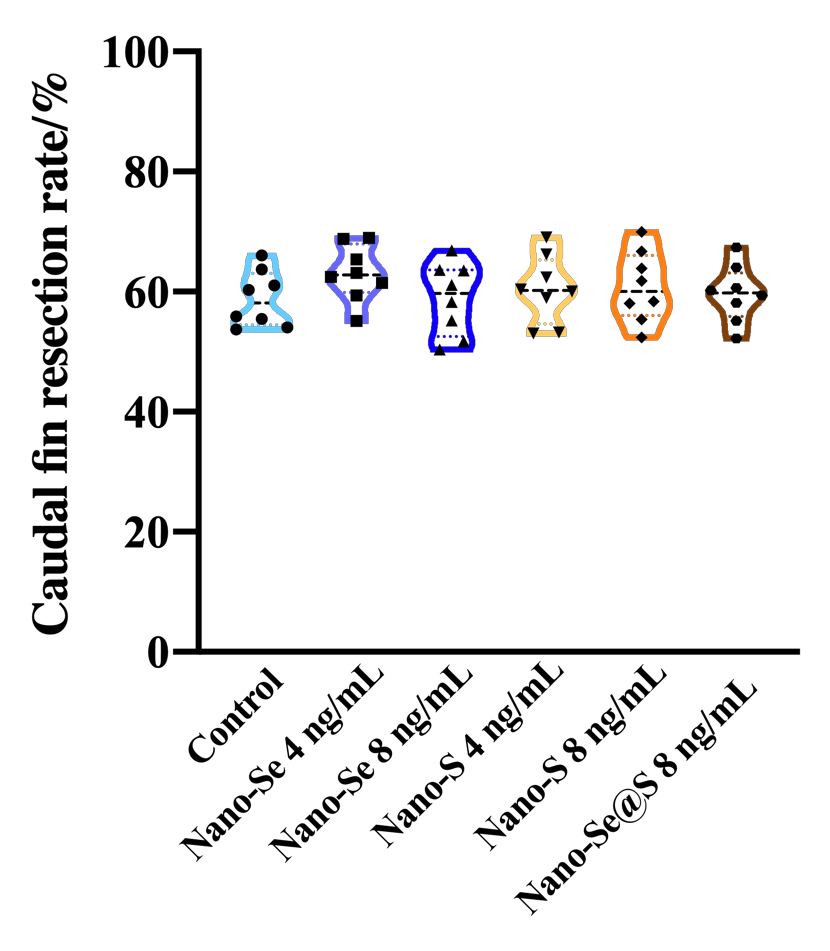
**

**Figure S1.** Caudal fin resection rate of zebrafish treated with Nano-Se, Nano-S, Nano-Se@S


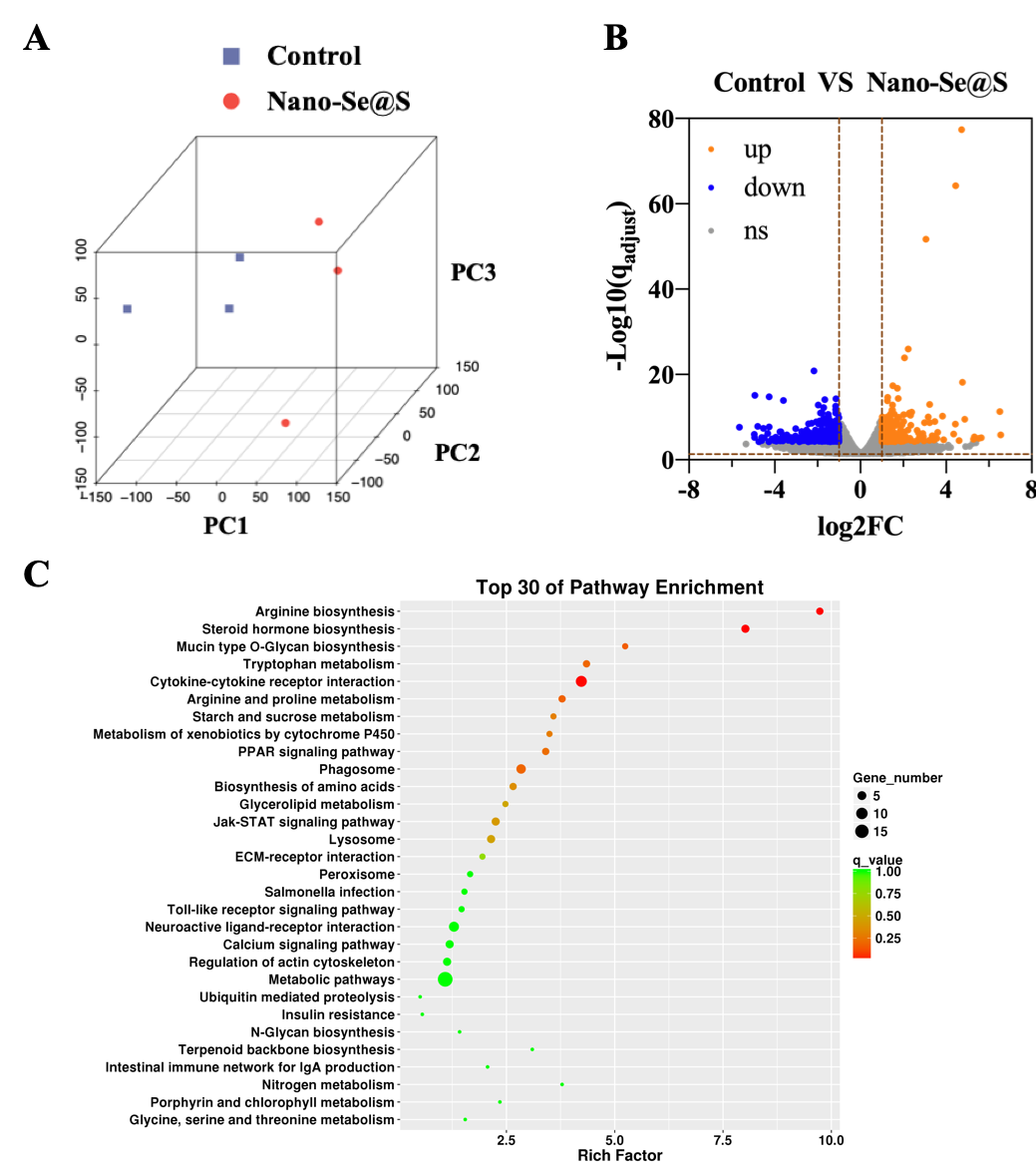


**Figure S2. (A)** Principal component analysis (PCA) was performed based on differentially expressed genes from regenerated tail fins of two groups. Each data point corresponds to the PCA analysis of each sample. **(B)** Volcano plots show the identified upregulated and downregulated genes by Nano-Se@S. **(C)** KEGG pathway enrichment analysis of the identified differentially expressed genes. The 30 most significantly enriched pathways are shown.


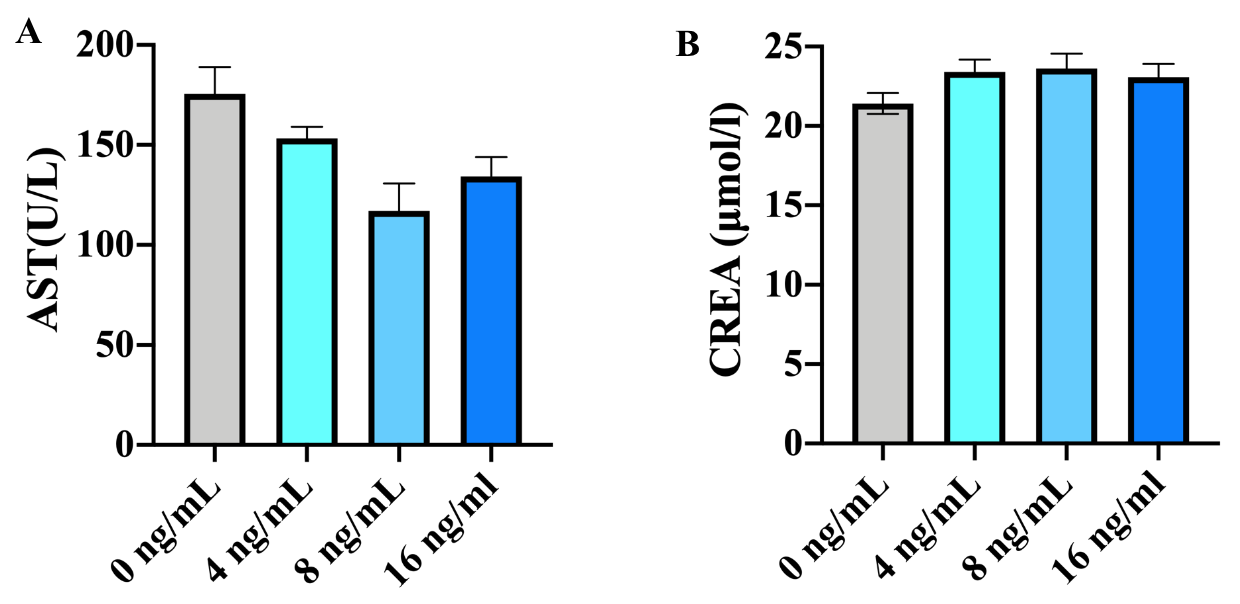


**Figure S3. (A)** Detection of AST index in mouse blood；**(B)** Detection of AST index in mouse blood.


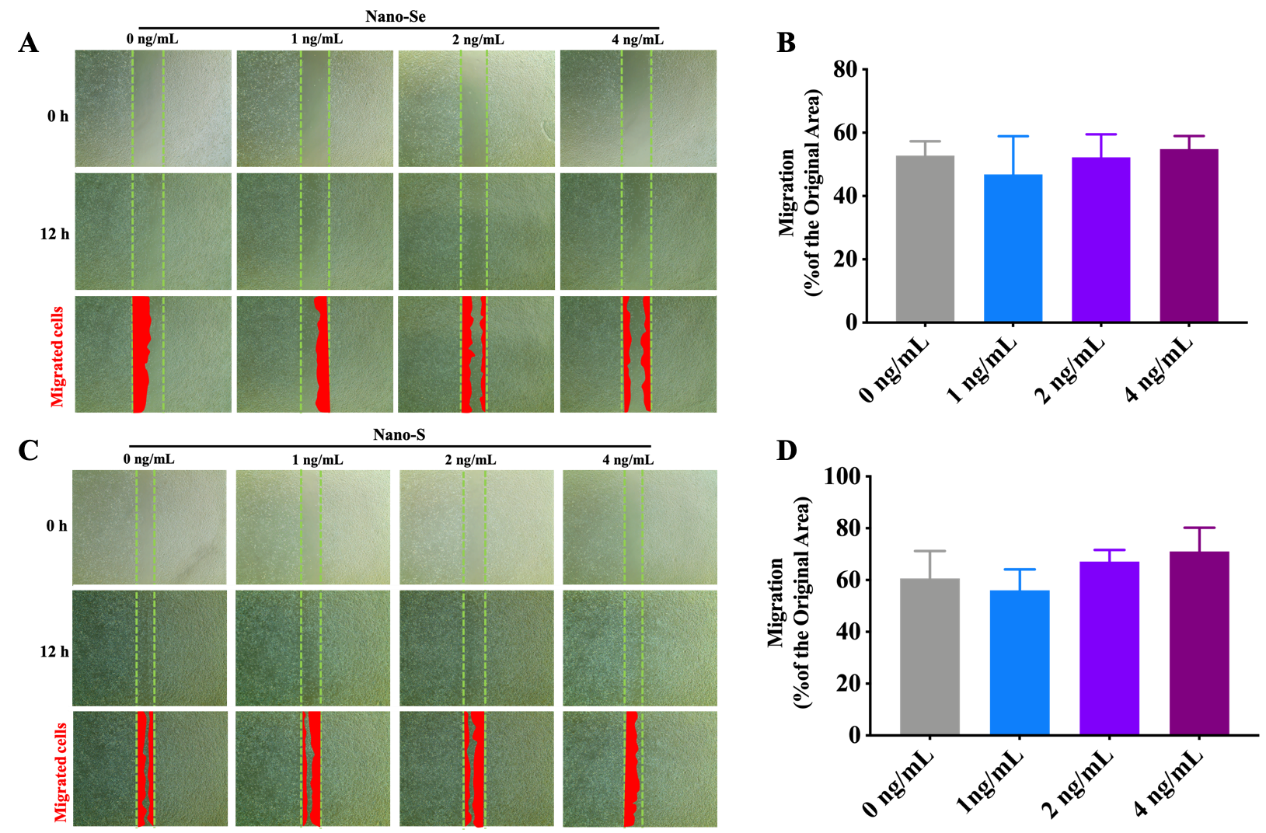


**Figure S4.** **(A)** Wound healing assay to evaluate the migration of HFF cells after being treated with Nano-Se and DMEM with 10% FBS. Cells were wounded and monitored using a microscope for 12 h. The red areas represent migrating cells. (**B)** The migration rate of HFF cells induced by Nano-Se. **(C)** Wound healing assay to evaluate the migration of HFF cells after being treated with Nano-S and DMEM with 10% FBS. Cells were wounded and monitored using a microscope for 12 h. The red areas represent migrating cells. (**D)** The migration rate of HFF cells induced by Nano-S (3 independent biological repeats n=9)


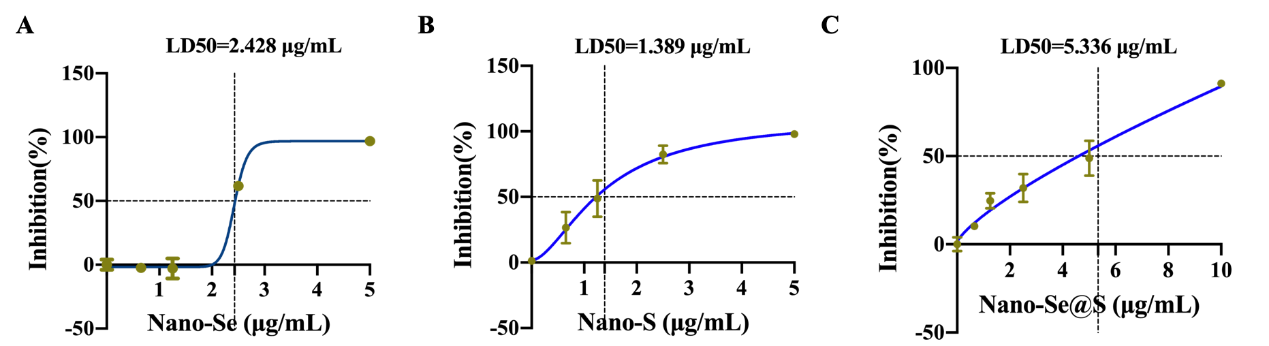


**Figure S5.** (A) The LD50 of the Nano-Se (0, 0.625, 1.25, 2.5, 5 μg/mL) in HFF cells by CCK8 assay; (B) The LD50 of the Nano-S (0, 0.625, 1.25, 2.5, 5 μg/mL) in HFF cells by CCK8 assay; (C) The LD50 of the Nano-S (0, 0.625, 1.25, 2.5, 5 ,10 μg/mL) in HFF cells by CCK8 assay. (n=3)


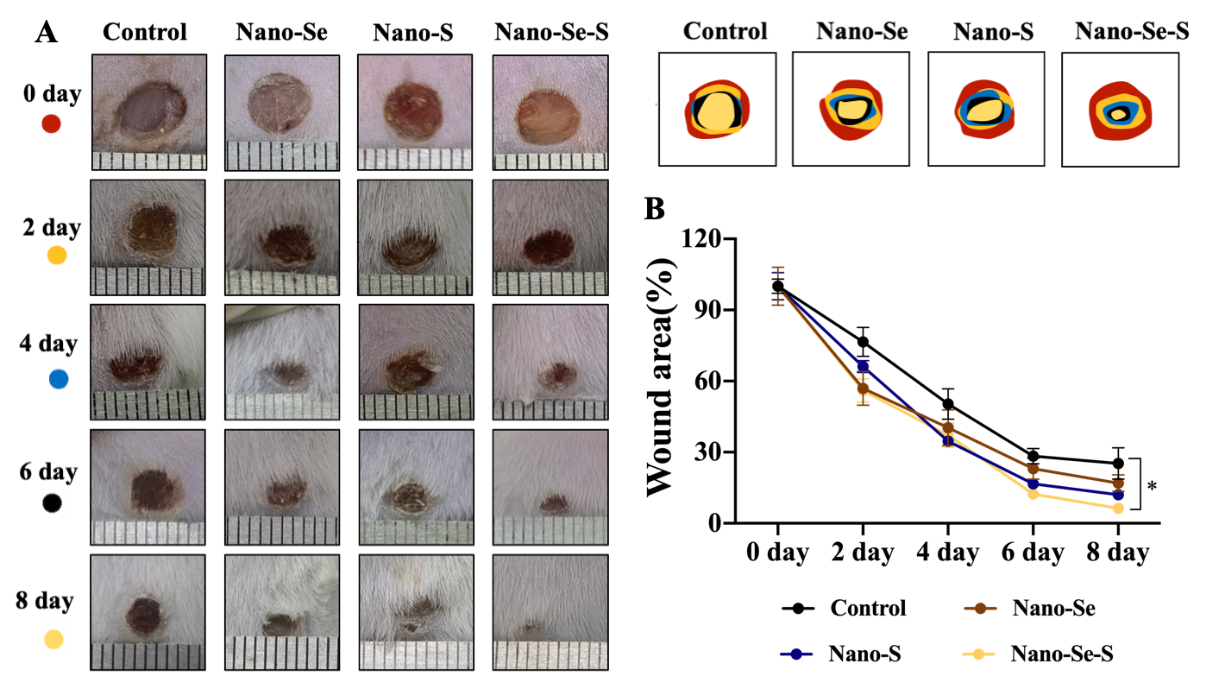


**Figure S6. (A)** The photographs of skin wound images treated with Nano-Se (4 ng/mL), Nano-S (4 ng/mL), Nano-Se@S (8 ng/mL). **(B)** Closed area ratio of skin wounds. (n=6, Mean values ± SD, *P < 0.05, **P < 0.01, ***P < 0.001, ****P < 0.0001)


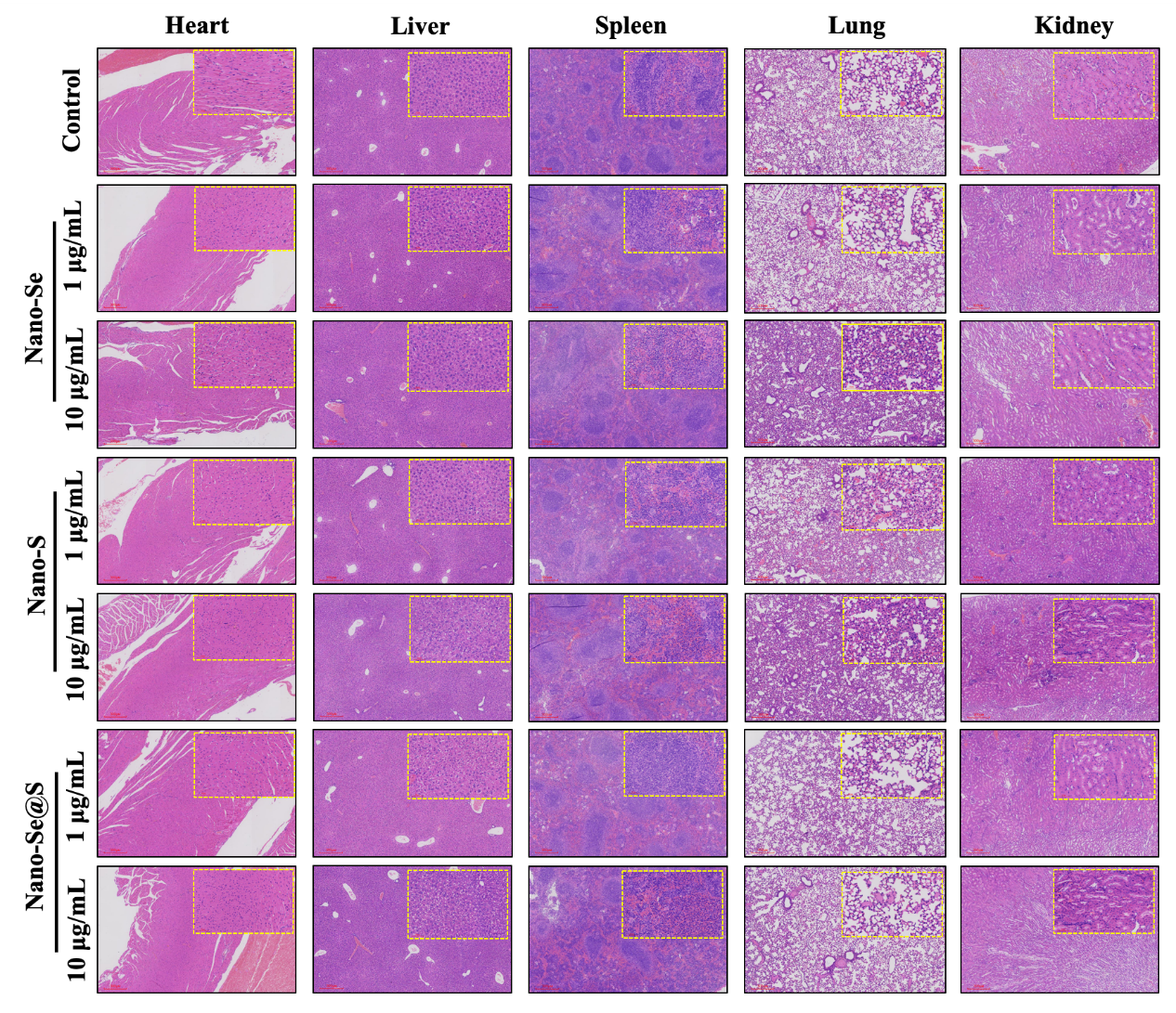


**Figure S7.** H&E staining of major organs (heart, liver, spleen, lung and kidney) showing the biosafety of different drug formulations, Scale bar is 300 μm. Yellow box: 5 times magnification image.
